# Association of depression and anxiety with cardiovascular co-morbidity in a primary care population in Latvia: a cross-sectional study

**DOI:** 10.1186/s12889-018-5238-7

**Published:** 2018-03-06

**Authors:** R. Ivanovs, A. Kivite, D. Ziedonis, I. Mintale, J. Vrublevska, E. Rancans

**Affiliations:** 1 Department of Psychiatry and Narcology, Riga Stradins University, 2 Tvaika Str, Riga, LV-1005 Latvia; 2 Department of Public Health and Epidemiology, Riga Stradins University, 9 Kronvalda Ave, Riga, LV-1010 Latvia; 3 Associate Vice Chancellor for Health Sciences, University of California San Diego, Biomedical Sciences Building, Room 1310, 9500 Gilman Drive #0602, La Jolla, CA 92093 USA; 4 Department of Cardiology, University Clinic of Paul Stradins, 13 Pilsonu Str, Riga, LV-1002 Latvia

**Keywords:** Depression, Anxiety, Cardiovascular disease, Latvia

## Abstract

**Background:**

Cardiovascular (CV) diseases (CVDs) are the leading cause of mortality worldwide. Globally, there is a growing interest in understanding and addressing modifiable psychosocial risk factors, particularly depression and anxiety, to prevent CVDs and to reduce morbidity and mortality. Despite the high premature mortality rate from CVDs in Latvia, this is the first Latvian study to examine the association of depression and anxiety with CVD morbidity in a primary care population.

**Methods:**

This cross-sectional study was carried out in 2015 within the framework of the National Research Program BIOMEDICINE at 24 primary care facilities throughout Latvia. Consecutive adult patients during a one-week time period at each facility were invited to join the study. Assessments onsite included a 9-item Patient Health Questionnaire (PHQ-9) and a 7-item Generalized Anxiety Disorder scale (GAD-7) followed by a socio-demographic questionnaire and measurements of height, weight, waist circumference, blood pressure, and total cholesterol. The diagnostic Mini International Neuropsychiatric Interview (MINI) was conducted over the telephone within 2 weeks after the visit to the general practitioner. A multivariate model was developed using binary logistic regression.

**Results:**

From the 1565 subjects (31.2% male), CVD was detected in 17.1%. Depression screening was positive (PHQ-9 ≥ 10) for 14.7%, and anxiety screening was positive (GAD-7 ≥ 10) for 10.1% of the study subjects. According to the MINI, 10.3% had current and 28.1% had lifetime depressive episode, and 16.1% had an anxiety disorder. Depression, not anxiety, was statistically significantly related to CVDs with an odds ratio (OR) of 1.52 (*p* = 0.04) for current depressive symptoms (PHQ-9 ≥ 10) and 2.08 (*p* = 0.002) for lifetime depressive episode (MINI).

**Conclusions:**

Current depressive symptoms (PHQ-9 ≥ 10) and a lifetime depressive episode (according to the MINI) were significantly associated with increased risk of CV morbidity. Therefore, CV patients should be screened and treated for depression to potentially improve the prognosis of CVDs. Enhanced training and integration of mental health treatment in Latvian primary care settings may improve clinical outcomes.

## Background

Anxiety and major depressive disorder (MDD) are the most common mental disorders in the European Union (EU) affecting 20.9% of the population (99.4 million people every year). MDD is already now the most important single contributor to the total disease burden not only in the EU but also worldwide, as measured by disability-adjusted life years (DALYs) [1, 2]. Anxiety disorders are the most frequent mental disorders in the general population with a prevalence rate of 14% [1]. Mental disorders, particularly anxiety and depression, are highly prevalent in patients with chronic somatic illnesses, affecting approximately 50% of patients in primary care settings [3, 4], and they are associated with poorer prognosis and increased treatment non-compliance, financial costs, other resource utilization, lost productivity and disability [5, 6]. The comorbidity of depression, anxiety and cardiovascular (CV) diseases (CVDs) is an especially important public health concern because CVDs are the leading cause of death globally, representing 31% of all deaths [7]. In Latvia, the mortality rate from CVDs is one of the highest in the EU, reaching 57% of all death in 2015 [8, 9], with the standard premature mortality from CVDs three times higher than on average than in the EU [10].

Depression and anxiety are highly prevalent in patients with CVDs [11]. Approximately one in five patients hospitalized for an acute coronary event meets the diagnostic criteria for MDD, and about half (40–65%) demonstrate sub-syndromal depressive symptoms [12], a prevalence rate that is at least three times higher than in the general population [13]. This proportion is even greater in stroke survivors, affecting nearly one in three patients [14, 15]. Clinically significant symptoms of anxiety have been reported in 20% to 42% of the CVD population [16–18].

Over the last 25 years, a large body of evidence has demonstrated that depression and anxiety are not only more common in CV patients, but that these two psychiatric conditions are also risk factors for increased cardiac morbidity and recurrent CV events and mortality, independent of traditional CV risk factors [19, 20]. The seminal INTERHEART study, involving 15,152 myocardial infarction (MI) cases from 52 countries, revealed that psychosocial factors such as depression and anxiety account for 32% of the population attributable risk (PAR) for MI, a level of risk comparable to that of smoking (PAR, 35.7%) and even greater than that of diabetes (PAR, 9.9%) and hypertension (PAR, 17.9%) [21]. Depression predicts incident coronary heart disease (CHD) (relative risk (RR) = 1.9 (95% confidence interval (CI): 1.49–2.42)) [22] and stroke (hazard ratio (HR) = 1.45 (95% CI: 1.29–1.63)) [23]. Previous meta-analyses evaluating the prognostic association of depression with mortality and new CV events in patients with already established CVDs demonstrated that depression is associated with a 1.6- to 2.6-fold increased risk of future major adverse cardiovascular events, cardiac mortality and all-cause mortality [24, 25], a level similar to traditional CV risk factors such as reduced left ventricular ejection fraction and diabetes [26]. A meta-analysis by Roest et al. in 2010 summarizing 20 prospective studies found that anxiety was associated with a 26% increased risk of incident CHD (HR = 1.26; 95% CI: 1.15–1.38) and a 48% increased risk of cardiac mortality (HR = 1.48; 95% CI: 1.14–1.92), independent of demographic variables, biological risk factors, and health behaviors [27]. A more recent meta-analysis that included 37 studies with 1,565,699 participants found an even stronger association and showed that anxiety was associated with a 52% increased incidence of CVD (HR = 1.52, 95% CI: 1.36–1.71) [28], despite some studies pointing to a possible protective role of anxiety in CVD prognosis [29, 30]. Another recent meta-analysis by Emdin et al. in 2016 concluded that the association of anxiety with stroke was stronger (RR = 1.71, 95% CI: 1.18–2.50) than the association of anxiety with CHD (RR = 1.41, 95% CI: 1.23–1.61) [31].

The largest cross-sectional surveys on CV risk factors in the EU called European Action on Secondary and Primary Prevention by Intervention to Reduce Events (EUROASPIRE) were conducted to determine whether the Joint European Societies guidelines on CVD prevention are being followed in clinical practice [32, 33]. EUROASPIRE III and IV surveys concluded that large proportions of CV patients in the EU do not achieve lifestyle, risk factor and therapeutic targets and there is considerable variations between European countries in patients’ CV risk factor prevalences and use of cardioprotective medications [34, 35]. The Baltic States (Estonia, Latvia and Lithuania) appeared to be among the most profoundly CVD-affected countries within Europe with very high CV mortality rates compared to other countries in the EU [10, 34]. It was explained by high prevalence of CV risk factors (dyslipidemia, obesity, diabetes and hypertension) that may relate to a larger proportion of poorer and older people, patients with lower education, as well as those outside social support networks, problems in doctor-patient relationship, inadequate dosing of drugs, unhealthy lifestyle [36, 37]. Comorbidity of depression and/or anxiety with CVD as a possible explanation has not been sufficiently examined in the Baltic region. Search of the literature revealed only few studies from Lithuania and Estonia that provided conflicting results [38, 39]. As the premature mortality rate from CVD in Latvia is alarmingly high [10], this topic is of particular importance for Latvia where there have been no studies to date.

We therefore aimed to conduct the first study on the association of depression and anxiety with CVDs in a primary care population in Latvia. This study addresses an important gap in the literature by focusing on Latvia. Recent evidence reported major cross-country differences in the determinants of disability among patients with heart disease and supported implementation of country-specific programs to reduce disability among CV patients [40]. Therefore local data are crucially important for the management of CV patients and for medical and nursing education, policy, and program development in Latvia.

## Methods

The cross-sectional study was carried out in 2015 within the framework of the National Research Program BIOMEDICINE 2014–2017 to assess the prevalence of the most frequent mental disorders in primary care settings in Latvia. Patients were recruited from 24 primary care facilities all over the country (16 in urban and 8 in rural areas). The survey was conducted in Latvian and Russian (the two most commonly spoken languages in Latvia).

### Ethics

This study was approved by the Ethics Committee of the Riga Stradins University, Riga, Latvia (No. 8/ 18.06.2015.). The project was carried out in accordance with the Declaration of Helsinki and its subsequent amendments. All respondents were enrolled only after providing written informed consent.

### Subjects and procedures

The inclusion criteria were consecutive treatment-seeking patients visiting a general practitioner (GP), patients aged 18 or older, and patients who had provided their informed consent. The exclusion criteria were patients who refused to participate in the study, patients younger than 18 years of age, and patients with acute medical condition requiring urgent hospitalization.

During a one-week period at each primary care facility, all consecutive patients who corresponded to the inclusion criteria were invited to complete a nine-item Patient Health Questionnaire (PHQ-9) and a seven-item Generalized Anxiety Disorder scale (GAD-7) in Latvian or Russian (language as preferred by participant) followed by an interview with a structured socio-demographic questionnaire and measurements of height, weight, waist circumference, blood pressure and total cholesterol on the same study visit. The Mini International Neuropsychiatric Interview (MINI) was conducted over the telephone by specially trained psychiatrists within a period of two weeks after the first contact. Diagnoses of CVD were confirmed using medical records. CVD was defined as an atherosclerotic vascular disease in the heart (CHD, angina, MI), brain (cerebrovascular disease, transient ischemic attack, stroke) and periphery (peripheral arterial disease), or a combination of these conditions [19]. Additionally, information about the diagnosis of depression and anxiety disorders, prescription of cardiovascular and psychotropic medications, and blood test results of lipids, glucose and glycated hemoglobin from the previous 3 months were obtained from medical documents.

### Assessment tools and measures

The PHQ-9 is a self-rating tool that was developed from the Primary Care Evaluation of Mental Disorders (PRIME-MD) questionnaire in the late 1990s to screen and to diagnose patients with depressive disorders in primary care settings [41]. It consists of nine items that correspond to the Diagnostic and Statistical Manual of Mental Disorders, Fourth Edition (DSM-IV) diagnostic criteria for MDD [42]. Respondents rate the questionnaire items from 0 to 3 according to the frequency of their experience over the previous two-week period (not at all, several days, more than half the days, or nearly every day). A systematic review [43] of the diagnostic accuracy of most widely used screening tools for depression among patients with chronic physical health problems found that the PHQ-9 with a cut-off score equal to or greater than 10 has good sensitivity (84%) and specificity (88%) to detect MDD and is even superior to the Hospital Anxiety and Depression Scale (HADS) (sensitivity = 75%; specificity = 81%). The first large-scale UPBEAT-UK study of the accuracy of commonly used depression screening measures within a primary care CHD population also concluded that the PHQ-9 appeared diagnostically superior when compared to HADS [44]. The Latvian and Russian versions of the PHQ-9 for Latvia were validated as a part of the National Research Project BIOMEDICINE 2014–2017 [45, 46].

The GAD-7 is a self-reported measure of anxiety [47, 48]. Participants were asked to consider the preceding two weeks and to rate symptom frequency as not at all (0), several days (1), more than half of all days (2) or nearly all days (3). Scores of 5, 10, and 15 were taken as the cut-off points for mild, moderate and severe anxiety, respectively. Using the threshold score of 10, the GAD-7 has a sensitivity of 89% and a specificity of 82% for generalized anxiety disorder. It is moderately good at screening three other common anxiety disorders: panic disorder (PD) (sensitivity = 74%, specificity = 81%), social anxiety disorder (SAD) (sensitivity = 72%, specificity = 80%) and post-traumatic stress disorder (PTSD) (sensitivity = 66%, specificity = 81%) [49]. A recent psychometric analysis carried out by Conway et al. also supported the reliability and validity of the GAD-7 in cardiac patients [50].

The MINI is a short, structured, diagnostic interview that was developed for DSM-IV and the psychiatric disorders portion of the 10th version of the International Classification of Diseases (ICD-10). It was validated in relation to the SCID-P (Structured Clinical Interview for DSM-III-R Patient Version) and the CIDI (Composite International Diagnostic Interview) [51]. It consists of 120 questions and screens 17 axis I disorders for 24 current and lifetime diagnoses. In this study MINI interview was used to identify current and/or lifetime episodes of major depressive disorder and most common anxiety disorders (generalized anxiety disorder, panic disorder, agoraphobia, social phobia, obsessive-compulsive disorder and post-traumatic stress disorder) [1]. The MINI was previously translated into 67 languages, including Latvian and Russian, according to standard methodology (forward/backward translation and clinician review) [52].

The structured questionnaire contained questions about demographics (age, gender, ethnicity, marital status, employment status, education), the medical reason for a visit to the general practitioner, CV risk factors, history of CVDs, diabetes mellitus and psychiatric disorders, use of CV and psychotropic medications, and days absent from work during last 3 months.

Assessment of CV risk factors was based on recommendations and criteria of the 2012 Joint European Societies’ guidelines on cardiovascular disease prevention [33] and questionnaires of the Finbalt Health Monitor System survey [53]. The following criteria were used for CV risk factors definitions:

1. Family history of premature CVD was defined as a fatal or non-fatal CVD event or/and established diagnosis of CVD in first degree male relatives before 55 years of age or female relatives before 65 years of age.
2. The question about leisure-time physical activity was: “How often do you do physical exercise at leisure lasting at least 30 min. making you at least mildly short of breath or perspire?” It was categorized as follows: 1) Unable to perform; 2) 1 time a week or less; 3) 2–3 times a week; 4) 4–6 times a week; 5) Every day.
3. The variable describing the prevalence of smoking was based on several questions regarding different aspects of smoking (smoking status, smoking history, number of cigarettes, last time smoked). It was categorized as follows: 1) Ever smoked and 2) Never smoked.
4. Alcohol consumption representing episodes of heavy drinking was measured with the following question: “Have you used 5 or more doses of alcohol at once during the last 12 months?” It was dichotomized as follows: 1) Yes and 2) No. Dose of alcohol was defined as equal to 10 mL by volume of 8 g by weight of pure alcohol.
5. The question about the use of fresh vegetables and fruits was: “Do you consume at least 200 g of fresh vegetables or/and fruits per day?” And it was categorized as follows: 1) Yes and 2) No.
6. Consumption of fish was considered as a protective factor if patient responded positively to the following question: “Do you consume fish at least 2 times per week (one of which to be oily fish)?”

### Statistical analysis

Statistical Package for the Social Sciences (SPSS) version 20.0 (IBM SPSS Corp.) was used for all statistical analyses. Statistical significance was evaluated at the level of *p* < 0.05. Crude and stratified percentages were used for descriptive statistics. To identify factors associated with the presence of CVDs, univariate and multivariate analyses were carried out. The multivariate model was developed by using binary logistic regression. To avoid multi-collinearity, separate regression models were elaborated for two indicators of depression as independent predictors of CVD- lifetime depressive episode (identified by the MINI interview) and current depressive symptoms (detected through the PHQ-9 instrument). For the final model of multivariate analysis, generalized anxiety disorder (according to the MINI) was chosen, as it gave the best-fitting regression model when compared to other anxiety disorders identified by the MINI or to clinically relevant anxiety symptoms detected by the GAD-7 questionnaire.

## Results

### Description of the study sample

The mean study response rate was 91.3% (from 1756 approached patients 152 refused to participate), it varied in the range between 86.3–93.7% across 24 primary care facilities all over the country. Those who refused did not significantly differ in the basic socio-demographic characteristics from the rest of the group. In this study, 1604 patients were invited to complete the PHQ-9 and the GAD-7 questionnaires, and 1585 of the approached patients completed both questionnaires. For those who completed both questionnaires, information about the presence of CVD diagnosis was available in 1565 subjects, 489 (31.2%) men and 1076 (68.8%) women, which were included in the final analysis. CVD (angina, myocardial infarction, stroke, transient ischemic attack, chronic cerebrovascular disorder and/or peripheral arterial disease) was detected in 17.1% (*n* = 268) of the studied population. The prevalence of CVD was slightly higher among men than women, with values of 18.4% and 16.5%, respectively.

One third (31.2%) of the study sample were males, and one third (28.8%) had a university degree. Slightly more than a half (55.8%) of the respondents had reached or exceeded the age of 55 years, and half (51.9%) were employed. One fifth (19.9%) of the study subjects were residing in the capital city, Riga (Table 1).

**Table 1:** Description of the study sample; prevalence of cardiovascular diseases in subgroups of the independent variables

Independent variable	CVD positive		CVD negative		Total	
	n	%	n	%	n	%
Gender						
Female	90	18.4	399	81.6	489	31.2
Male	178	16.5	898	83.5	1076	68.8
Age						
≤ 54 years	15	2.2	677	97.8	692	44.2
≥ 55 years	253	29.0	620	71.0	873	55.8
Education						
9-years basic and unfinished basic education	58	27.2	155	72.8	213	13.7
General or vocational secondary and unfinished secondary education	147	16.5	746	83.5	893	57.5
Higher and unfinished higher education	61	13.6	387	86.4	448	28.8
Employment status						
Economically inactive	208	31.5	452	68.5	660	42.4
Unemployed	5	5.7	83	94.3	88	5.7
Employed	54	6.7	754	93.3	808	51.9
Place of residence						
Riga	111	35.7	200	64.3	311	19.9
Other city	121	16.2	627	83.8	748	47.8
Rural	36	7.1	470	92.9	506	32.3
Positive family history of premature cardiovascular disease (< 55 years in men and < 65 years in women)						
Unknown	16	24.2	50	75.8	66	4.2
Yes	96	16.2	497	83.8	593	38.1
No	155	17.3	743	82.7	898	57.7
Smoking status						
Ever smoked	79	14.5	465	85.5	544	35.0
Never smoked	188	18.6	824	81.4	1012	65.0
Alcohol use, episodes of heavy drinking in last 12 months (5 or more doses of alcohol at once)						
Yes	23	9.0	232	91.0	225	16.4
No	244	18.7	1058	81.3	1302	83.6
Consumption of fresh vegetables and fruits ≥ 200 g per day (2–3 servings)						
Yes	187	16.2	968	83.8	1155	74.2
No	80	19.9	322	80.1	402	25.8
Consumption of fish ≥ 2 times per week, one of which to be oily fish						
Yes	108	17.6	505	82.4	613	39.4
No	159	16.8	785	83.2	944	60.6
Sedentary lifestyle (30 min. of moderate physical activity)						
Unable to perform	50	29.1	122	70.9	172	11.1
1 time a week or less	94	13.9	580	86.1	674	43.5
2–3 times a week	34	16.4	173	83.6	207	13.4
4–6 times a week	20	11.2	158	88.8	178	11.5
Every day	68	21.3	251	78.7	319	20.5
Body mass index, kg/m^2^						
≤ 24.99 (normal + underweight)	62	13.0	415	87.0	477	30.8
≥ 25.00 (overweight + obese)	203	19.0	868	81.0	1071	69.2
Diabetes mellitus						
Yes	42	29.6	100	70.4	142	9.1
No	226	15.9	1197	84.1	1423	90.9
Total cholesterol, mmol/l						
< 5 (normal)	133	22.0	471	78.0	604	39.2
5+ (increased)	131	14.0	807	86.0	938	60.8
Systolic hypertension, mm Hg						
< 140 (below)	134	15.1	755	84.9	889	57.4
140+ (hypertension)	131	19.9	528	80.1	659	42.6
Diastolic hypertension, mm Hg						
< 90 (below)	196	18.4	870	81.6	1066	68.9
90+ (hypertension)	69	14.3	413	85.7	482	31.1
Anxiety (MINI, any anxiety disorder)						
Yes	37	15.9	196	84.1	233	16.1
No	209	17.2	1007	82.8	1216	83.9
Anxiety (GAD-7)						
Yes	30	19.2	126	80.8	156	10.1
No	234	16.8	1156	83.2	1390	89.9
Agoraphobia (MINI)						
Yes	19	16.2	98	83.8	117	8.1
No	227	17.0	1105	83.0	1332	91.9
Generalized anxiety disorder (MINI)						
Yes	16	18.0	73	82.0	89	6.1
No	230	16.9	1130	83.1	1360	93.9
Panic disorder (MINI)						
Yes	1	9.1	10	90.9	11	0.8
No	245	17.0	1193	83.0	1438	99.2
Depression (PHQ-9)						
Yes	58	25.4	170	74.6	228	14.7
No	206	15.6	1112	84.4	1318	85.3
Current depression (MINI)						
Yes	34	22.8	115	77.2	149	10.3
No	212	16.3	1088	83.7	1300	89.7
Lifetime depression (MINI)						
Yes	80	19.7	327	80.3	407	28.1
No	166	15.9	876	84.1	1042	71.9
Antihypertensive medications						
Yes	226	29.4	542	70.6	768	49.1
No	42	5.3	755	94.7	797	50.9
Cholesterol lowering medications						
Yes	117	49.2	121	50.8	238	15.2
No	151	11.4	1176	88.6	1327	84.8
Antidepressants						
Yes	10	21.3	37	78.7	47	3.0
No	258	17.0	1260	83.0	1518	97.0

A positive family history of premature CVD was reported in 38.1% of respondents. At the time of the questionnaire, one third (35.0%) of the study subjects were tobacco smokers, 16.4% had 5 or more drinks of alcohol at least once during the preceding year, one quarter (25.8%) did not include fresh fruits and vegetables in their meals daily, and 60.6% reported that they did not eat fish regularly (i.e., at least twice per week). Daily moderate physical activity was reported by one fifth (20.5%) of individuals. More than two thirds (69.2%) of the patients were overweight according to BMI, and more than half had increased levels of total cholesterol (60.8%). The prevalence of diabetes among the respondents was 9.1%. Systolic or diastolic hypertension were present in 42.6% and 31.1% of patients, respectively. Thus, at the time of the questionnaire, 49.1% of the individuals were taking antihypertensive medications, and 15.2% were taking cholesterol lowering medicines (Table 1).

As shown in Table 1, anxiety disorders (according to the MINI) were identified in 16.1% of patients (8.1% had agoraphobia, 6.1% had generalized anxiety disorder and 0.8% had panic disorder). Anxiety screening (by using the GAD-7 instrument) was positive for one tenth (10.1%) of the study subjects. Current depressive symptoms (PHQ-9 ≥ 10) were present in 14.7% (*n* = 228) of the individuals. According to the MINI questionnaire, 10.3% (*n* = 149) had current and 28.1% (*n* = 407) had lifetime depressive episode. Antidepressants were used by 3.0% of individuals, which could have been for depression or anxiety disorders.

### Factors associated with CVD

In the univariate analyses, the factors statistically significantly associated with the presence of CVD (i.e., increasing the odds of CVD) were older age, lower education, economical inactivity, urban place of residence, smoking status, episodes of heavy drinking, being overweight, the presence of diabetes, systolic hypertension, depression and intake of antihypertensive or cholesterol lowering medications (Table 2).

**Table 2:** Factors associated with CVD in univariate and multivariate analyses

Independent variable	OR	95% CI	*p*	aOR1*	95% CI	*p*	aOR2**	95% CI	*p*
Gender									
Male vs. female	1.14	0.86–1.51	0.37	1.37	0.87–2.15	0.17	1.35	0.87–2.11	0.18
Age									
≥ 55 years vs. ≤ 54 years	18.42	10.82–31.36	< 0.001	5.29	2.80–9.99	< 0.001	5.46	2.87–10.38	< 0.001
Education									
9-years basic / unfinished basic vs. higher and unfinished higher	2.37	1.58–3.56	< 0.001	1.65	0.94–2.91	0.08	1.55	0.87–2.74	0.13
General / vocational secondary, unfinished secondary vs. higher / unfinished higher	1.25	0.91–1.73	0.18	1.11	0.73–1.70	0.63	1.03	0.67–1.58	0.89
Employment status									
Economically inactive vs. employed	6.43	4.66–8.86	< 0.001	2.39	1.55–3.69	< 0.001	2.28	1.47–3.52	< 0.001
Unemployed	0.84	0.33–2.16	0.71	1.18	0.38–3.62	0.78	1.25	0.40–3.88	0.70
Place of residence									
Riga vs. rural	7.25	4.81–10.93	< 0.001	9.22	5.31–16.04	< 0.001	8.98	5.16–15.63	< 0.001
Other city vs. rural	2.52	1.70–3.72	< 0.001	3.26	1.98–5.36	< 0.001	3.08	1.87–5.07	< 0.001
Positive family history of premature cardiovascular disease (< 55 years in men and < 65 years in women)									
Unknown vs. no	1.53	0.85–2.76	0.16	1.09	0.47–2.53	0.85	0.97	0.42–2.26	0.95
Yes vs. no	0.93	0.70–1.22	0.59	1.16	0.79–1.69	0.45	1.12	0.77–1.63	0.57
Smoking status									
Ever vs. never	0.75	0.56–0.99	0.04	1.15	0.73–1.82	0.53	1.17	0.75–1.85	0.49
Alcohol use, episodes of heavy drinking in last 12 months (5 or more doses of alcohol at once)									
Yes vs. no	0.43	0.27–0.68	< 0.001	0.72	0.38–1.37	0.32	0.71	0.37–1.35	0.29
Consumption of fresh vegetables and fruits ≥ 200 g per day (2–3 servings)									
Yes vs. no	1.29	0.96–1.72	0.09	0.90	0.60–1.35	0.61	0.87	0.58–1.31	0.51
Consumption of fish ≥ 2 times per week, one of which to be oily fish									
Yes vs. no	0.95	0.72–1.24	0.69	1.01	0.71–1.45	0.94	1.00	0.70–1.44	1.00
Sedentary lifestyle (30 min. of moderate physical activity)									
Unable to perform vs. every day	1.51	0.99–2.31	0.06	0.69	0.38–1.23	0.21	0.67	0.37–1.21	0.19
1 time a week or less vs. every day	0.60	0.42–0.85	0.004	0.62	0.39–0.99	0.04	0.60	0.38–0.96	0.03
2–3 times a week vs. every day	0.73	0.46–1.14	0.17	0.87	0.48–1.59	0.65	0.88	0.48–1.62	0.69
4–6 times a week vs. every day	0.47	0.27–0.80	0.005	0.63	0.30–1.30	0.21	0.62	0.30–1.29	0.20
Body mass index, kg/m^2^									
≥ 25.00 (overweight + obese) vs. ≤ 24.99 (normal + underweight)	1.57	1.15–2.13	0.004	0.86	0.56–1.32	0.49	0.86	0.56–1.33	0.51
Diabetes mellitus									
Yes vs. no	2.23	1.51–3.28	< 0.001	0.96	0.57–1.63	0.89	0.94	0.56–1.60	0.83
Total cholesterol, mmol/l									
5+ (increased) vs. < 5 (normal)	0.58	0.44–0.75	< 0.001	0.76	0.52–1.10	0.14	0.77	0.53–1.12	0.17
Systolic hypertension, mm Hg									
140+ (hypertension) vs. < 140 (below)	1.40	1.07–1.82	0.01	1.03	0.69–1.54	0.87	0.98	0.65–1.46	0.91
Diastolic hypertension, mm Hg									
90+ (hypertension) vs. < 90 (below)	0.74	0.55–1.00	0.05	0.96	0.61–1.45	0.77	0.94	0.60–1.46	0.77
Generalized anxiety disorder (MINI)									
Yes vs. no	1.08	0.62–1.88	0.80	0.75	0.36–1.56	0.44	0.64	0.30–1.38	0.25
Anxiety (GAD-7)									
Yes vs. no	1.18	0.77–1.79	0.45	1.19***	0.67-2.12	0.55	0.89***	0.49-1.63	0.71
Lifetime depression (MINI)									
Yes vs. no	1.29	0.96–1.74	0.09	1.52	1.02–2.25	0.04	–	–	–
Depression (PHQ-9)									
Yes vs. no	1.84	1.32–2.57	< 0.001	–	–	–	2.08	1.30–3.32	0.002
Antihypertensive medications									
Yes vs. no	7.50	5.30–10.61	< 0.001	3.45	2.16–5.49	< 0.001	3.37	2.11–5.36	< 0.001
Cholesterol lowering medications									
Yes vs. no	7.53	5.55–10.22	< 0.001	2.99	1.99–4.50	< 0.001	3.01	2.00–4.54	< 0.001
Antidepressants									
Yes vs. no	1.32	0.65–2.69	0.44	1.51	0.60–3.80	0.38	1.63	0.66–4.07	0.29

*aOR1 – adjusted odds ratio, 1st model (lifetime depression according to MINI included)

**aOR2 – adjusted odds ratio, 2nd model (current depression according to PHQ-9 included)

***Adjusted for all variables except generalized anxiety disorder (MINI) to avoid multi-collinearity

After adjustment, only 6 out of the mentioned 11 factors remained significant predictors of CVD. Older age had one of the strongest associations with the CVD, and it increased the odds approximately five times (*p* < 0.001). Economic inactivity (including pensioners, disabled persons, housewives, students) doubled the odds of having a CVD (*p* < 0.001). Residence in the capital city (Riga) increased the risk of CVD approximately nine times (*p* < 0.001), and living in another urban area increased the odds more than 3 times (*p* < 0.001) when compared to living in a rural area (Table 2). Individuals who performed moderate physical activity of 30 min once a week or less had decreased odds of having CVD compared to individuals who reported physical activity every day (*p* = 0.03). Patients who were using antihypertensive or cholesterol lowering medications had three times higher odds of having a CVD (*p* < 0.001).

The odds ratio (OR) for current depressive symptoms (according to the PHQ-9) was 1.50 (*p* = 0.046) and for lifetime depressive episode (detected by the MINI) the OR was 2.11 (*p* = 0.002) (Table 2). The current depressive episode according to the MINI did not show statistically significant results in the univariate analysis or any multivariate model. None of the anxiety measures (MINI and GAD-7) showed statistically significant associations with CVD in either the univariate or the multivariate analyses.

Gender stratified analysis of study population showed that current depressive symptoms (according to the PHQ-9) were associated with 2.04 (*p* = 0.004) higher odds of having CVD in women, but not in men (data not shown).

## Discussion

To the best of our knowledge, this is the first study in Latvia that explores the association between CVD and depression and anxiety in the primary care population. The main findings of this cross-sectional study were that individuals with current depressive symptoms (PHQ-9 ≥ 10) demonstrated 2.08 (95% CI: 1.30–3.32, *p* = 0.002) times higher odds of having a CVD, and a lifetime depressive episode according to the MINI was associated with an adjusted OR for CVD of 1.52 (95% CI: 1.02–2.25, *p* = 0.04).

### Association of depression and CVDs

An overview of 59 prognostic studies from 3 meta-analyses demonstrated that depression was associated with a 1.5- to 2.7-fold increased risk of incident CVD [24, 54]. A more recent and updated meta-analysis by Gan et al. reviewed 30 prospective studies (*n* = 893,850) published up to April 2014 with a follow-up duration ranging from 2 to 37 years. They found a more modest association of depression, with CHD and MI reporting pooled RRs of 1.30 (95% CI: 1.22–1.40) and 1.30 (95% CI: 1.18–1.44), respectively [20]. Due to the cross-sectional nature of this study, we are not able to draw conclusions about causality of the established link between depression and CVDs. But our findings in the context of previous research suggest that depression might be a risk factor for increased CV morbidity in Latvian population that has to be investigated in future prospective studies. Although we found a cross-sectional relationship between the current depressive symptoms (PHQ-9 ≥ 10) and lifetime depressive episode according to the MINI, unexpectedly there was no statistically significant association with a diagnosis of current depressive episode detected with the structured psychiatric interview (MINI). Our finding could be explained in two ways. First, 97 individuals of our study population who completed the PHQ-9 questionnaire were not interviewed with the MINI, so this omission could have attenuated the statistical power of the MINI results. Second, the MINI is a categorically based depression assessment tool that could have excluded individuals with sub-syndromal depressive symptoms from case status. On the other hand, the PHQ-9 is a dimensional assessment tool, which could have allowed the inclusion of people with clinically significant depressive symptoms who failed to meet the formal criteria for DSM IV and ICD-10 diagnosis. However, the existing evidence showed that sub-threshold depressive symptoms were significantly associated with increased disability, morbidity and mortality [55]. Therefore, we consider the statistically significant association of the PHQ-9 score ≥ 10 with CVD in the Latvian primary care population as a clinically relevant finding and an easily assessed marker for primary care providers to target.

Evidence about the comorbidity of depression and CVD in other Baltic countries is contradictory. A cross-sectional study in Lithuania involving 317 individuals from primary care centers in 20 cities investigated the association between psychosocial stress, manifested as anxiety and depression, and CVD using the Hospital Anxiety and Depression Scale (screening tool for detection of elevated anxiety and depressive symptoms) [38]. Burokiene et al. demonstrated a modest but significant correlation between CVD and current depressive symptoms (OR = 1.18; 95% CI: 1.07–1.31, *p* = 0.001) but found no statistically important correlation between current anxiety symptoms and CVD. On the other hand, a cross-sectional study involving 1094 patients from 23 primary care practices across Estonia that explored which co-morbid diseases are associated with depression (using the Depression Section of the Composite International Diagnostic Interview) did not confirm higher co-morbidity of CVD in depressed patients when compared to non-depressed individuals [39].

Specific genetic, behavioral and pathophysiological factors have been established as contributing to the initiation, progression, and clinical manifestation of athero-thrombotic CVD in those suffering from depression and anxiety disorders. Behavioral factors are related to an unhealthy lifestyle (smoking, excessive alcohol consumption, unhealthy diet, sedentary behavior) and poor treatment adherence (medical treatment regimen, maintaining smoking cessation, participation in cardiac rehabilitation) [56–60]. Pathophysiological factors worsening CVD include dysregulation in the autonomic nervous system and hypothalamic-pituitary-adrenal axis as well as metabolic and immuno-inflammatory dysregulations. These pathophysiological factors can lead to coronary vasoconstriction, hypertension, left ventricular hypertrophy, reduced heart rate variability, endothelial dysfunction, platelet activation, hypercoagulability, and the production of pro-inflammatory cytokines (C-reactive protein, interleukin-6, intercellular adhesion molecule-1). These changes elevate the risk of ventricular arrhythmias, MI and stroke [61–63]. We examined whether our finding of the association of depression and CVD could be explained by other common traditional and non-traditional CV risk factors, including smoking, exercise, body mass index, and alcohol consumption [22]. In their meta-analysis, Nicholson et al. reported that only half (10 out of 21) of the studies evaluated this range of CV risk factors [22]. In the logistic regression analysis of our study, the association between depressive measures and CVD morbidity remained significant even after adjustment for the traditional CV risk factors.

### Association of anxiety and CVDs

In contrast to depression, which in numerous studies has been linked to the increased morbidity and mortality of CVDs, less is known about the influence of anxiety symptoms and anxiety disorders. The most recent meta-analysis, summarizing a total of 37 studies including 1,565,699 participants, demonstrated a 52% increased risk of CVD onset (HR = 1.52, 95% CI: 1.36–1.71) for patients with anxiety symptoms and disorders. This finding seemed to be independent of traditional CV risk factors and depression [28]. The most extensively studied anxiety disorders associated with the onset and progression of CVDs, adverse cardiovascular outcomes, including mortality, are generalized anxiety disorder, panic disorder and post-traumatic stress disorder [64]. Patients with GAD suffer from excessive anxiety and worry, accompanied by somatic anxiety symptoms as well as from restlessness, irritability, difficulty concentrating, muscle tension, sleep disturbances and being easily fatigued. Panic disorder is characterised by recurrent panic attacks, which are discrete periods of intense fear or discomfort, accompanied by various somatic and psychic symptoms. PTSD develops after a terrifying ordeal that involved physical harm or the threat of physical harm and is characterised by recurrent and intrusive distressing recollections of the event, nightmares, dissociative flashback episodes, distress at exposure to cues that resemble the traumatic event, avoidance of stimuli associated with the trauma, estrangement from others, sleep disturbances, irritability, difficulty concentrating, hypervigilance and exaggerated startle response [65, 66]. However, in contrast, some studies reported beneficial effects of anxiety on CV morbidity and mortality [29, 30]. These studies proposed left ventricular function as an important factor that may modulate the prognostic significance of anxiety and may improve risk stratification in CV patients.

We have not found a statistically significant association of GAD (detected by the MINI) with CVD (*p* = 0.25). This could be explained by the insufficient sample size of our study population. Only 89 individuals corresponded to the MINI diagnostic criteria of GAD. Similarly, a previously mentioned cross-sectional study in the primary care population of Lithuania (*n* = 317) by Burokiene et al. also reported no significant correlation between anxiety and CVD [38]. There is a need for larger studies with increased numbers of patients conducted in Latvia and the Baltic region to explore the association of anxiety and CVD morbidity with sufficient statistical power.

### Association of other CV risk factors and CVDs

Several reports have shown that some well-established CV risk factors, such as tobacco smoking, heavy drinking and sedentary lifestyle [19], may be associated with major depression and anxiety disorders [67–70]. Although the univariate analysis of our study demonstrated relationships between these risk factors and CVD, after adjustment, only sedentary lifestyle maintained a statistically significant association with CVD. It was found in our study that moderate physical activity once a week or less decreased the odds of CVD when compared to everyday activity. This might be explained by the fact that individuals who are performing physical activity more frequently are more likely to have been detected as having a higher risk for CVD and thus have received a strong recommendation from the primary care physician to practice a health-promoting lifestyle. Our data analysis shows a tendency for some binge drinking episodes within the last 12 months to be preventive in relation to CVD when compared with absence of alcohol abuse. This might be because the population of non-abusers partially consists of people who have stopped their heavy drinking habit due to some cardiovascular health threats. In future studies, it might be prudent to use more sensitive instrument for alcohol consumption that takes into account not only recent but also lifetime habits.

Another new finding was the highest adjusted odds ratio of 8.98 (95% CI: 5.16–15.63, *p* < 0.001) for individuals living in the urban area of Riga (capital of Latvia) compared to those living in rural areas. This result seems to be consistent with the data obtained in the Prospective Urban Rural Epidemiologic cohort study involving more than 150,000 adults in 17 high-, middle-, and low-income countries [71]. Yusuf et al. (2014) reported that urban communities had a higher CV risk-factor burden than rural communities but had lower rates of CV events and lower case fatality rates. Our study adds to this finding by Yusuf et al. and other similar studies that reported the significance and relevance of considering the place of residence (urban versus rural) as an important moderator and/or confounding factor to include in future studies on CV risks and outcomes [22, 27].

The EUROASPIRE studies have shown that effectiveness of CV risk factors management in the EU is still far from optimal and that major cross-country differences exist [34, 35]. The Baltic countries are among the most profoundly CVD-affected countries with higher CV mortality rates compared to other countries in the EU [10]. Local data are very important to develop country-specific management programs for CV patients [40]. However, previous largest studies on CV risk factors prevalence in Latvian population have not assessed depression and anxiety as possible risk factors [36, 72, 73]. Findings of this study suggest that detection and treatment of depression should be included in CV patients` management programs and future research on CV risk factors in Latvia.

### Strengths and limitations

The strengths of this study include the use of a diagnostic interview for the diagnosis of anxiety disorders, a sample that includes persons from a wide range of ages, and a hierarchical multivariate analysis that includes adjustment for anxiety and multiple traditional CV and socio-economic risk factors, including place of residence, which has rarely been used in previous studies. Another strength is that only specially trained psychiatrists were involved in data collection.

The results of this study must be considered in the context of some limitations. Although this is a large, nationally representative primary care convenience sample survey that provides comprehensive data, due to the cross-sectional design of this study, we are not able to draw unequivocal conclusions about the direction of the identified associations between depression or anxiety and CVD morbidity. Second, the PHQ-9 using the standard cut-point of ≥ 10 has been recognized as a reliable tool for detecting depression in chronic somatic conditions, including CVD, and has been found to be even superior to other commonly used screening instruments [43, 44]. However, the PHQ-9 is not a standardized criterion for the diagnosis of depression, which may lead to false positive results. Moreover, there is an ongoing discussion about the optimal PHQ-9 thresholds specific for patients with CVD, with some studies indicating that the PHQ-9 cut-off score of ≥8 or even ≥6 would improve the sensitivity and specificity of this instrument [44, 74]. Further studies are needed to clarify the optimal PHQ-9 threshold for the Latvian CVD population. Third, there is a need for increased numbers of patients to improve the statistical significance of our findings.

## Conclusions

We have found a statistically significant association between current depressive symptoms (PHQ-9 ≥ 10) and lifetime depressive episode (according to the MINI) with increased risk of CVD morbidity; however, the same association was not found for anxiety disorders or current anxiety symptoms. Living in an urban area, especially in the capital city, has been established as an important associated factor that was rarely used in previous research. The findings of this study suggest that CV patients should be screened and treated for depression to potentially improve the prognosis of CVD. Enhanced training and integration of mental health treatment in Latvian primary care settings may improve clinical outcomes. Further research with a larger sample size should be undertaken to better assess the effect of anxiety on CVD morbidity and to investigate the optimal PHQ-9 threshold for the Latvian CVD population.

## Abbreviations

CHD: Coronary heart disease
CI: Confidence interval
CIDI: Composite International Diagnostic Interview
CV: Cardiovascular
CVD: Cardiovascular disease
DSM-IV: Diagnostic and Statistical Manual of Mental Disorders, Fourth Edition
EU: European Union
GAD: Generalized anxiety disorder GAD-7 Generalized Anxiety Disorder questionnaire, 7
GP: General practitioner
HADS: Hospital Anxiety and Depression Scale
HbA1c: Glycated hemoglobin
HR: Hazard ratio
ICD-10: 10th version of the International Classification of Diseases
MDD: Major depression
MI: Myocardial infarction
MINI: Mini International Neuropsychiatric Interview
OCD: Obsessive-compulsive disorder
OR: Odds ratio
PAR: Population attributable risk
PD: Panic disorder
PHQ-9: Patient Health Questionnaire, 9
PTSD: Post-traumatic stress disorder
RR: Relative risk
SAD: Social anxiety disorder
SCID-P: Structured Clinical Interview for DSM-III-R Patient Version
SPSS: Statistical package for the Social Sciences
